# From bench to bedside: in vitro and in vivo evaluation of a neonate-focused nebulized surfactant delivery strategy

**DOI:** 10.1186/s12931-019-1096-9

**Published:** 2019-07-02

**Authors:** F. Bianco, F. Ricci, C. Catozzi, X. Murgia, M. Schlun, A. Bucholski, U. Hetzer, S. Bonelli, M. Lombardini, E. Pasini, M. Nutini, M. Pertile, S. Minocchieri, M. Simonato, B. Rosa, G. Pieraccini, G. Moneti, L. Lorenzini, S. Catinella, G. Villetti, M. Civelli, B. Pioselli, P. Cogo, V. Carnielli, C. Dani, F. Salomone

**Affiliations:** 1 Department of Preclinical Pharmacology, R&D, Chiesi Farmaceutici S.p.A, Parma, Italy; 2 Scientific Consultancy, Saarbrücken, Germany; 3 PARI Pharma GmbH, Starnberg, Germany; 4 Division of Neonatology, Cantonal Hospital Winterthur, Winterthur, Switzerland; 5 Pediatric Research Institute “Città della Speranza”, Padova, Italy; 6 Mass Spectrometry Center (CISM), Polo Biomedico, Careggi University Hospital of Florence, Florence, Italy; 7 Health Science and Technologies Interdepartmental Center for Industrial Research (HST-ICIR), University of Bologna, Bologna, Italy; 8 Division of Pediatrics, Department of Medicine, University of Udine, Udine, Italy; 9 Polytechnic University of Marche and Azienda Ospedaliero-Universitaria Ospedali Riuniti, Ancona, Italy; 10 Department of Neurosciences, Psychology, Drug Research and Child Health, University of Florence School of Medicine, Careggi University Hospital of Florence, Viale Morgagni, 85 Florence, Italy

**Keywords:** Nebulized surfactant, *Poractant alfa*, eFlow-Neos, Respiratory distress syndrome, CPAP, Neonatal ventilation, Nebulizer

## Abstract

**Background:**

Non-invasive delivery of nebulized surfactant has been a neonatology long-pursued goal. Nevertheless, the clinical efficacy of nebulized surfactant remains inconclusive, in part, due to the great technical challenges of depositing nebulized drugs in the lungs of preterm infants. The aim of this study was to investigate the feasibility of delivering nebulized surfactant (*poractant alfa*) in vitro and in vivo with an adapted, neonate-tailored aerosol delivery strategy.

**Methods:**

Particle size distribution of undiluted *poractant alfa* aerosols generated by a customized eFlow-Neos nebulizer system was determined by laser diffraction. The theoretical nebulized surfactant lung dose was estimated in vitro in a clinical setting replica including a neonatal continuous positive airway pressure (CPAP) circuit, a cast of the upper airways of a preterm neonate, and a breath simulator programmed with the tidal breathing pattern of an infant with mild respiratory distress syndrome (RDS). A dose-response study with nebulized surfactant covering the 100–600 mg/kg nominal dose-range was conducted in RDS-modelling, lung-lavaged spontaneously-breathing rabbits managed with nasal CPAP. The effects of nebulized *poractant alfa* on arterial gas exchange and lung mechanics were assessed. Exogenous alveolar disaturated-phosphatidylcholine (DSPC) in the lungs was measured as a proxy of surfactant deposition efficacy.

**Results:**

Laser diffraction studies demonstrated suitable aerosol characteristics for inhalation (mass median diameter, MMD = 3 μm). The mean surfactant lung dose determined in vitro was 13.7% ± 4.0 of the 200 mg/kg nominal dose. Nebulized surfactant delivered to spontaneously-breathing rabbits during nasal CPAP significantly improved arterial oxygenation compared to animals receiving CPAP only. Particularly, the groups of animals treated with 200 mg/kg and 400 mg/kg of nebulized *poractant alfa* achieved an equivalent pulmonary response in terms of oxygenation and lung mechanics as the group of animals treated with instilled surfactant (200 mg/kg).

**Conclusions:**

The customized eFlow-Neos vibrating-membrane nebulizer system efficiently generated respirable aerosols of undiluted *poractant alfa*. Nebulized surfactant delivered at doses of 200 mg/kg and 400 mg/kg elicited a pulmonary response equivalent to that observed after treatment with an intratracheal surfactant bolus of 200 mg/kg. This bench-characterized nebulized surfactant delivery strategy is now under evaluation in Phase II clinical trial (EUDRACT No.:2016–004547-36).

**Electronic supplementary material:**

The online version of this article (10.1186/s12931-019-1096-9) contains supplementary material, which is available to authorized users.

## Background

Current trends in the management of preterm infants with Respiratory Distress Syndrome (RDS) aim for gentler, non-invasive interventions as first-line treatment options. Over the last years, the classic ways of providing artificial respiratory support, oxygen therapy, and exogenous surfactant have been systematically challenged [1–5] in order to reduce the iatrogenic effects that contribute to the development of chronic lung disease [6, 7]. Particularly, clinical research has been directed to reduce the use of mechanical ventilation [8–10], a major risk factor associated with lung inflammation and the development of Broncho-Pulmonary Dysplasia (BPD). Therefore, non-invasive ventilation techniques are gaining momentum as the primary treatment of RDS [10–13].

Surfactant replacement therapy has also undergone a clear evolution towards less invasive administration protocols [14]. Aerosol delivery of exogenous surfactant in combination with nCPAP has been proposed as a feasible, truly non-invasive surfactant delivery method [15, 16]. The theoretical advantages of nebulization include minimal manipulation of the respiratory tract, improved pulmonary distribution [17], and the avoidance of the acute airway fluid load occurring immediately after surfactant instillation [16]. A gradual surfactant administration by nebulization may reduce some of the side effects associated with surfactant instillation, e.g. transient airway obstructions and reflux, hypercarbia, and hypoxia [16], and may therefore contribute to more stable systemic and cerebral hemodynamics [18–20]. Experimental studies with aerosolized surfactant, however, have shown controversial results, ranging from no effect at all of the aerosolized surfactant to an equivalent performance compared to intratracheal bolus instillation [17–30]. A few clinical studies have also attempted to deliver nebulized surfactant to preterm infants managed with CPAP [31–35]. These studies enrolled a limited number of patients, applied heterogeneous surfactant administration protocols, and used different nebulizers. So far, these clinical studies have demonstrated that nebulized surfactant is well tolerated and can be safely administered during non-invasive ventilation.

A great challenge in the field of aerosol delivery is to improve the extremely low lung deposition of inhaled drugs in preterm neonates, which has been reported to be lower than 1% of the nominal dose [36, 37]. Preterm neonates are forced nasal breathers, have a low functional residual capacity, high respiratory rate (RR), low tidal volume, and small airway caliber [38]. All these factors reduce the residence time of aerosol particles within the airways, which significantly reduces lung deposition. In addition, the bias flow of the ventilation support can dilute the concentration of surfactant droplets, leading to a relatively high surfactant loss through the expiratory limb of the CPAP circuit [38]. To overcome these limitations, research efforts have been directed to characterize, even further, the particular challenges posed by preterm infants to aerosol delivery, as well as to develop novel aerosol-generating devices and infant-focused strategies aimed at improving lung deposition of nebulized surfactant [39–45].

In the present work, we investigated the feasibility of delivering nebulized surfactant in vitro and in vivo with a customized, neonate-focused aerosol delivery strategy. For that purpose, we first analyzed the aerosol characteristics of nebulized *poractant alfa* (Curosurf®, Chiesi Farmaceutici SpA, Parma, Italy) generated by a customized eFlow-Neos vibrating-membrane nebulizer system (PARI Pharma, Starnberg, Germany) under physiological relative humidity (RH) conditions. We further investigated surfactant deposition in a realistic in vitro CPAP circuit, which included a cast of the upper airways of a preterm infant and a breath simulator programmed with a neonatal breathing pattern. In vitro data were used to implement surfactant nebulization in a nCPAP-supported RDS animal model, in which a dose-response study was performed to assess the pulmonary efficacy of nebulized surfactant.

## Materials and methods

### Surfactant particle size characterization

The particle size distribution of surfactant aerosols (*poractant alfa,* 80 mg/mL) generated by the customized eFlow-Neos nebulizer system was determined in vitro by laser diffraction (Helos/BF, Sympatec GmbH, Clausthal-Zellerfeld, Germany). Briefly, this technique measures the angular variation in the intensity of scattered light when a laser is directed through a particle dispersion. The angular variation in the intensity of scattered light is inversely proportional to particle size, which allows determining the size distribution of the particles in the surfactant cloud. To perform laser diffraction experiments, the customized eFlow-Neos was filled with 2 mL of undiluted surfactant that was then continuously nebulized towards the detection area. Laser diffraction experiments were conducted at 30% ± 5 or, alternatively, at 90% ± 5 relative humidity (RH) conditions, at 37 °C. Each of these experiments was repeated five times using independent nebulizer units. The mass median diameter (MMD), the geometric standard deviation (GSD), and the fine particle fraction (FPF) were used to characterize surfactant aerosols.

### Becnhmark breath simulation experiments

A set-up composed of a CPAP system (Fabian HFO, Acutronic, Zug, Switzerland) with a humidifier (MR 730, Fisher & Paykel Healthcare), a customized eFlow-Neos nebulizer system, a cast of the upper airways (nose-throat) of a premature infant (PrINT model) [41], infant nasal prongs (3520, Fisher & Paykel Healthcare), surfactant collection filters (PARI Filter PAD PZN: 00632160), and a breath simulator (Compas 2, PARI Pharma, Starnberg, Germany) [46, 47] was implemented in order to simulate a clinical setting of surfactant nebulization under non-invasive neonatal ventilation conditions. The PrINT model was developed by Minocchieri et al. by 3D reconstruction of a magnetic resonance of a premature infant born after 32 weeks of gestation (body weight of 1750 g) [41]. The infant nose-throat cast was 3D printed (1zu1 prototypen, Dornibirn, Austria) as a solid substance (material: DSM water clear ultra 10,122). The nose area was silicon-coated to achieve a tight connection between cast and prongs (Additional file 1: Figure S1).

The nebulizer was placed between the Y piece and the nasal prongs using a custom-made adaptor. Before surfactant nebulization, the set up was systematically checked for air leaks. The temperature of the system was 37 °C and the RH was set at 90% ± 5. Bias flow rate and CPAP level were set at 5 L/min and 5 cmH_2_O, respectively. The breath simulator was programmed with the following neonatal configuration: RR of 70 bpm, a tidal volume (V_T_) of 8.9 mL (5 mL/kg), and an inhalation/exhalation ratio of 40/60.

**Fig. 1:**
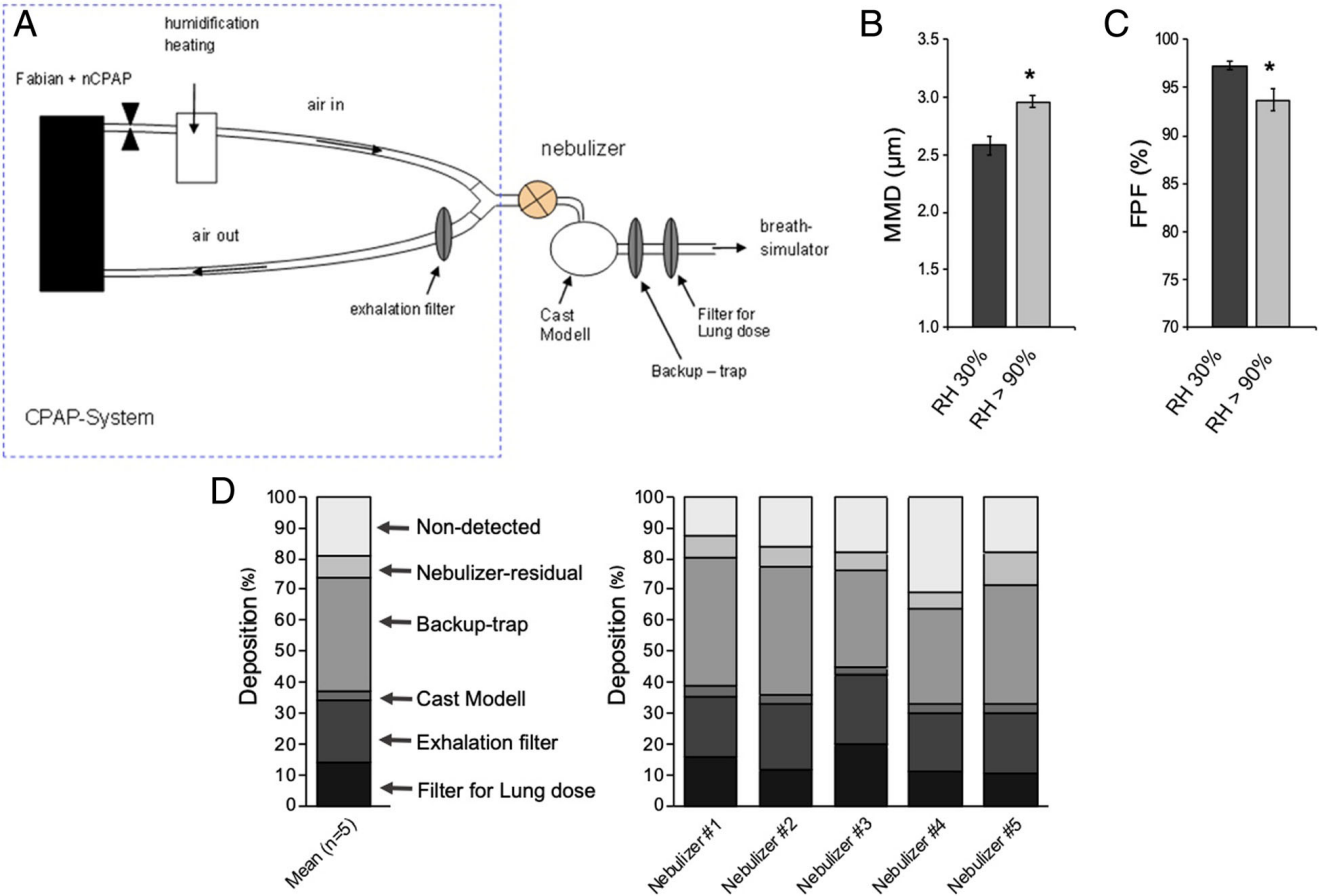
(**a**) Scheme of the experimental setup. (**b**) Mass median diameter (MMD) of nebulized surfactant measured by laser diffraction under different relative humidity (RH) conditions. (**c**) Fine particle fraction (FPF) of nebulized surfactant under different RH conditions. (**d**) Mean cumulative percentage of deposited surfactant within the different setup compartments (*n* = 5) and cumulative percentage of deposited surfactant for individual experiments, each of them conducted with independent nebulizer units. * *P* vs. RH 30% < 0.01

A volume of 4.37 mL of *poractant alfa* (80 mg/mL), the equivalent volume of a 200 mg/kg dose for a 1750 g infant, was loaded into the customized nebulizer reservoir and was continuously nebulized. Surfactant collection filters were placed in the expiratory limb of the CPAP system (exhalation filter) as well as at the distal airway of the PrINT cast (Filter for lung dose, Fig. 1a). Lung dose (LD) was defined as the amount of surfactant collected within the filter placed at the distal airway of the PrINT cast (In-Filter). A backup-trap was installed between the PrINT cast and the In-Filter in order to collect the surfactant liquid film formed from the already impacted aerosol particles. The backup-trap did not have a significant impact on the aerosol flow. The amounts of surfactant deposited in the CPAP circuit, exhalation filter, backup-trap, nasal prongs, and residual surfactant remaining in the nebulizer were also determined, after dissembling of the analytical setup. After nebulization of the full surfactant dose, the required nebulization time was noted. A rinsing solvent containing 50 mg of potasium nitrate and 85% v/v isopropanol was used to recover the surfactant from each compartment of the analytical set up. Samples for quantitative analysis were prepared by defined dilutions. The main active constituent of poractant alfa, i.e. Phosphatidylcholine (PC), was determined as lead compound in order to quantify the surfactant amount. A validated high-pressure liquid chromatography (HPLC) method using external standard calibration was used for quantifying PC distribution in the set-up compartments. The method is sensitive enough to determine PC in the applied concentration range from 20 to 2100 μg/ml for *poractant alfa* if a dual wavelength detector (WATERS 2487, Waters Corporation, Milford, US) is used.

### Animal preparation

The experiments were carried out in 6- to 7-week-old male rabbits (Charles River Laboratories, Calco, Italy). The experimental procedure was approved by the intramural Animal Welfare Body and the Italian Ministry of Health (Prot.n° 1300–2015-PR) and complied with the European and Italian regulations for animal care.

Rabbits (body weight of 1.5–2.5 kg) were initially sedated with intramuscular (i.m.) medetomidine (Domitor®, 2 mg/kg). The throat of the animals was first shaved and local anesthesia was applied in the anterior neck with lidocaine gel (Luan® 2.5%). Thirty minutes later, the animals received 50 mg/kg of ketamine (Imalgene 1000®, Merial-Boehringer Ingelheim, France) and 5 mg/kg of xylazine (Rompun®, Bayer, Germany) i.m. Rabbits, in the supine position, were intubated and stabilized on positive pressure ventilation (Fabian HFO, Acutronic, Zug, Switzerland) as previously described [48, 49]. Fraction of inspired oxygen (FiO_2_) = 100%, Flow = 10 L/min, RR = 40 breaths/min, positive end-expiratory pressure (PEEP) = 3 cmH_2_O, tidal volume (V_T_) targeted to 7 ml/kg (with the peak inspiratory pressure, PIP, not exceeding 15 cmH_2_O) and inspiratory time of 0.5 s. Airway flow, mean airway pressure (MAP) and V_T_ were monitored as long as the animals were intubated. Body temperature was constantly measured with a rectal probe and maintained at 37 degrees by placing a heating pad underneath the animal.

After endotracheal intubation, a catheter was inserted into the right jugular vein for continuous infusion of 1 mg/ml of ketamine and 0.1 mg/ml of xylazine in 0.9% saline solution (100 μl/min) to maintaining anesthesia at a steady level. Trometamol (tris-hydroxymethyl aminomethane, THAM, 1 M, Sigma-Aldrich, USA) was also infused during the surfactant depletion procedure for mantaing CO_2_ level under control. A second catheter was inserted into the right carotid artery for blood sampling. After instrumentation, baseline blood gases were measured with an emogas analyzer (Radiometer Medical, Denmark).

Animals with an initial arterial oxygen partial pressure (PaO_2_) value > 400 mmHg at PIP < 15 cmH_2_O were included in the study. Repeated broncho-alveolar lavages (BALs) were performed by flushing the airways with 20 ml/kg of pre-warmed 0.9% NaCl solution, followed by a short recovery period in-between, until a PaO_2_ value < 150 mmHg was reached. Then, if after 15 min of stabilization on mechanical ventilation the respiratory failure was confirmed (PaO_2_ < 150 mmHg, with PIP not exceeding 23 cmH_2_O), the animal was included in the study.

### Experimental groups

Forty-two animals were allocated to one of the six experimental groups. Animals in the **nCPAP group** (*n* = 6) were maintained in continuous nCPAP (Fabian HFO, Acutronic; 5 cmH_2_O) for 180 min, using customized nasal prongs as an interface. Animals allocated in the **Inst-SURF group** (*n* = 9) received an intratracheal bolus of *poractant alfa* (200 mg/kg), using a modified InSurE approach, and were further managed with nCPAP (5 cmH_2_O) for 180 min. After surfactant instillation, animals were managed with mechanical ventilation for 10 min (V_T_ = 7 mL/Kg, RR = 40/min and PEEP = 3 cmH_2_O). Animals receiving nebulized surfactant were allocated in four different groups of escalating surfactant doses. The customized eFlow-Neos nebulizer was placed between the Y piece and the customized nasal prongs. Rabbits in the **Neb-SURF100 group** (n = 9) received 100 mg/kg of nebulized *poractant alfa* while on nCPAP, whereas animals in the **Neb-SURF200** (n = 9), **Neb-SURF400 group** (*n* = 9), and **Neb-SURF600 group** (*n* = 9) received 200 mg/kg, 400 mg/kg, and 600 mg/kg of nebulized *poractant alfa*, respectively. All animals treated with nebulized surfactant were managed with nCPAP (5 cmH_2_O) for 180 min, counting from the initiation of surfactant nebulization. For each animal experiment, a new customized eFlow-Neos nebulizer unit was used. The time required to nebulize the whole surfactant dose was annotated.

### Gas exchange and respiratory indices

Arterial carbon dioxide partial pressure (PaCO_2_), PaO_2_, and pH were measured (Radiometer Medical, Denmark) right after the induction of anesthesia (baseline) and after the induction of respiratory distress. Arterial blood gases were also measured right after placing the animals on nCPAP, 15 and 30 min after the start of nCPAP, and then every 30 min until the end of the experiment.

PaO_2_ and PaCO_2_ were also used to compute the oxygenation index (OI) and the ventilation efficacy index (VEI). OI and VEI were determined at those time intervals in which the animals were ventilated with a tracheal tube: at baseline, after inducing the respiratory distress and at the end of the 180 min follow-up period. Therefore, after the 180 min observational period, animals were shifted from nCPAP to invasive mechanical ventilation for a brief period of time, using exactly the same ventilation settings as at baseline (FiO_2_ 100%, Flow = 10 L/min; RR = 40 bpm, PEEP = 3 cmH_2_O, V_T_ targeted to 7 ml/kg, and inspiratory time of 0.5 s). The OI was calculated as follows:

1 $$ \mathrm{OI}={\mathrm{FiO}}_2\ast \mathrm{MAP}\ast 100/{\mathrm{PaO}}_2 $$

The VEI was calculated to evaluate the overall ventilation efficiency of mechanically ventilated animals independently from the ventilation settings [50]:

2 $$ \mathrm{VEI}=3800/\left[\left(\mathrm{PIP}/\mathrm{PEEP}\right)\ast \mathrm{RR}\ast {\mathrm{PaCO}}_2\right] $$

Ventilation indices were calculated at baseline (pre-BALs), after inducing respiratory distress (post-BALs), and at the end of the observational period.

### Pulmonary mechanics

Dynamic compliance (C_dyn_) was also determined in those time intervals in which the animals were ventilated with invasive mechanical ventilation: at baseline, resembling the “healthy” pulmonary status, right after inducing severe respiratory distress by repeated BALs, and at the end of the observational period, after re-intubation. C_dyn_ was calculated by dividing lung volume (∆V, in mL) by the changes in pressure (∆P) standardized by the animal’s weight.

3 $$ {\mathrm{C}}_{\mathrm{dyn}}=\Delta  \mathrm{V}/\left(\Delta  \mathrm{P}\ast \mathrm{Weight}\right) $$

### Exogenous alveolar disaturated-phosphatidylcholine (DSPC) quantification

Intrapulmonary levels of exogenous surfactant were determined by administering *poractant alfa* labelled with U^13^C-PA-DPPC (*n* = 3 animals for each group). For tracer preparation, 0.2 mg (0.1 μl) of a suspension of U^13^C-PA-DPPC (Avanti Polar Lipids) in saline solution was carefully mixed with 200 mg (2.5 ml) of *poractant alfa,* which was then administered to distressed rabbits as described in the previous section. At the end of the experiment, BALs from all animal groups were collected and stored at − 80 °C. Lipids were extracted according to the method described by Bligh and Dyer [51]. Disaturated-phosphatidylcholine (DSPC) was separated by thin layer chromatography after treatment with osmium tretaoxide [52]. Fatty acids of DSPC were derivatized as methyl ester by adding 2 mL 3 M HCl methanol and extracted with hexane. Quantitative analysis of DSPC was performed by a FID gas chromatograph (GC, Agilent 5890, Milan, Italy). The ^13^C enrichment of DSPC from the BALs was measured with gas chromatography-mass spectrometry (GC-MS, Agilent, 5973 Inert), as described elsewhere [53]. For data display, the intrapulmonary exogenous surfactant amount of the animals treated with nebulized surfactant was normalized taking the surfactant instillation group as reference.

### Data analysis

In vitro and in vivo data are presented as mean ± SD. Raw data were analyzed and compared by repeated measures two-way analysis of variance (ANOVA) as a function of group and time, followed by Dunnett’s and Tukey’s t posthoc tests. Statistical analysis was performed using GraphPad software, version 6.0. A *P* < 0.05 was considered statistically significant.

## Results

### Particle size characterization of nebulized surfactant

Under low RH conditions (30% ± 5), the mean MMD of nebulized surfactant was 2.6 ± 0.1 μm. Increasing RH close to lung physiological conditions (90% ± 5) produced a slight, although significant, increase of the mean MMD to 3.0 ± 0.1 μm (Fig. 1b). Irrespective of RH conditions, all tested customized eFlow-Neos nebulizers showed a reproducible performance in terms of particle size with a consistent GSD of 1.5, indicative of a heterodisperse aerosol yet with a narrow variability in terms of particle size distribution.

The slight increase of the MMD under high RH conditions was associated with a slight decrease of the FPF. This parameter represents the percentage of particles contained in the aerosol cloud with a diameter below 5 μm, also known as the respirable fraction. Mean FPF values determined under low and high humidity conditions were 97.2% ± 0.4 and 93.7% ± 1.1, respectively (Fig. 1c).

### Benchmark breath simulation experiments

The mean nebulization time to deliver 4.37 mL of surfactant (80 mg/mL) was 18.9 min (range 13.3–23.3 min), which corresponds to an aerosol production rate of 18.5 mg phospholipid/min (range 15.0–26.3 mg/min). The determined mean LD was 13.7% ± 4.0 (range 10.2–19.8%, Fig. 1d and e), which would yield an estimated intrapulmonary surfactant dose of around 27 mg/kg in vivo if a 200 mg/kg dose was delivered. A fraction of generated aerosol was deposited on the backup-trap (36.7% ± 5.3), whereas 20.3% ± 1.5 was detected in the exhalation filter of the CPAP circuit. Only 3.0% ± 0.6 of the nebulized surfactant deposited within the PrINT cast and 7.2% ± 2.1 was collected as a residual volume in the nebulizer chamber. Surfactant recovery was 81.0% ± 6.9. The undetected surfactant fraction may have remained within the circuit as non-deposited particles that were further released to the environment during set up disassembling.

### Gas exchange and respiratory indices

**Fig. 2:**
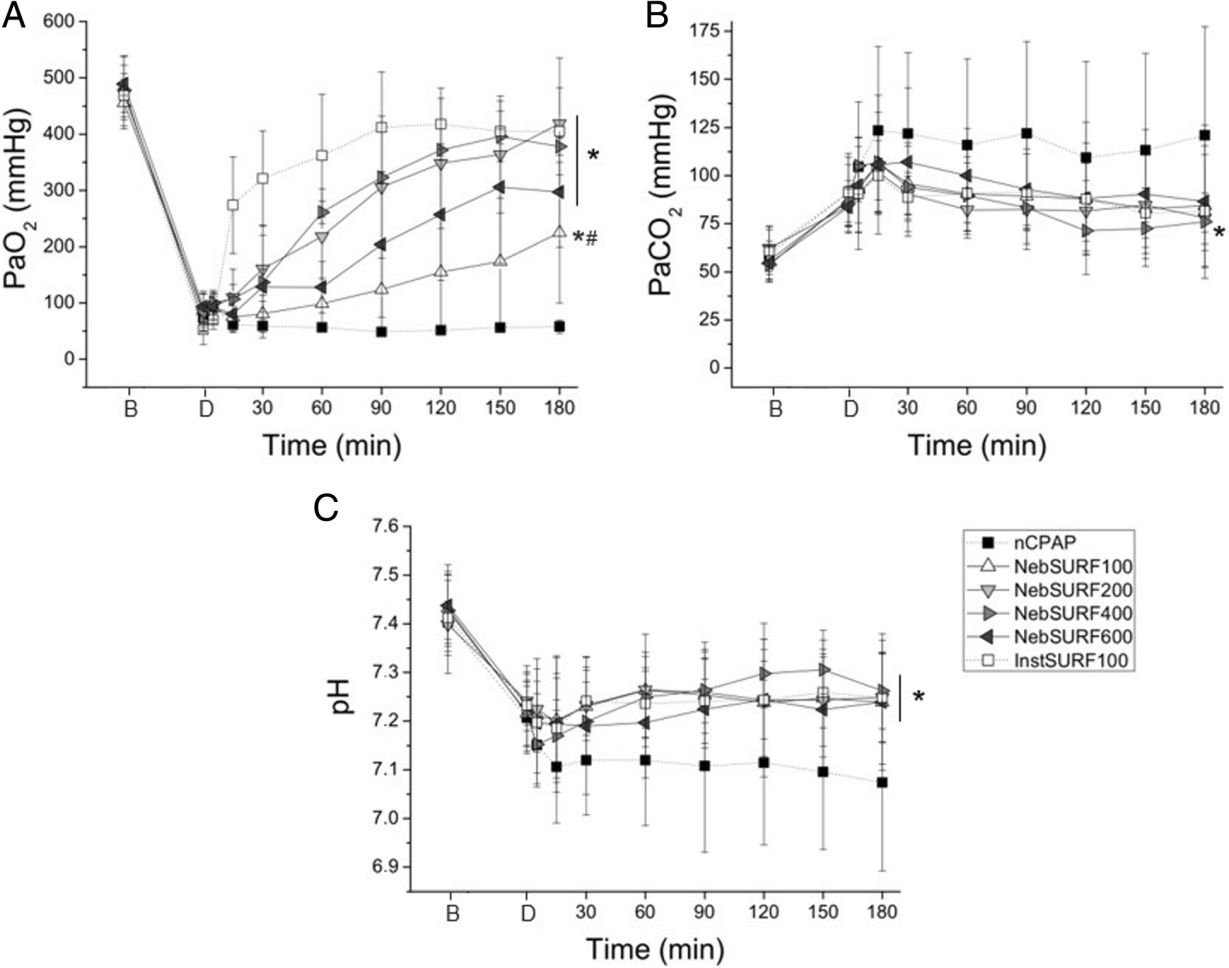
Mean PaO_2_ (**a**), PaCO_2_ (**b**), and pH (**c**) values over time in surfactant-depleted adult rabbits treated with nasal continuous positive pressure ventilation (nCPAP, black squares), with intratracheal surfactant (Inst-SURF, white squares), or with different doses of nebulized surfactant (Neb-SURF100, up-pointing triangles; Neb-SURF200, down-pointing triangles; Neb-SURF400, right-pointing triangles; and Neb-SURF600, left-pointing triangles). Values are shown as the mean ± SD. * *P* vs. nCPAP < 0.01; ^#^ *P* vs. Inst-SURF < 0.05

There were no significant differences between groups at baseline in terms of body weight, number of BALs needed to achieve the targeted respiratory failure, PaO_2_ or C_dyn_ (Additional file 1: Table S1). All animals had similar mean PaO_2_ at baseline (> 450 mmHg). However, following surfactant-depletion, a severe respiratory failure developed in all groups (mean PaO_2_ < 150 mmHg). The PaO_2_ in the nCPAP group remained extremely low, below 100 mmHg, despite a FiO_2_ of 100%. PaO_2_ values rapidly increased right after surfactant instillation, reaching mean PaO_2_ values above 200 mmHg 15 min after treatment (Fig. 2a). PaO_2_ values remained significantly higher throughout the whole experimental period in the Inst-SURF group compared to the nCPAP group (*P* < 0.01). In the groups of animals treated with nebulized surfactant, PaO_2_ increased gradually over time achieving significantly higher values compared to untreated control animals (*P* < 0.01). However, whereas mean PaO_2_ values of Neb-SURF200 and Neb-SURF400 groups were equivalent to the mean PaO_2_ of the Inst-SURF group at 180 min, the mean PaO_2_ of the Neb-SURF100 group was significantly lower (*P* < 0.01) compared to the latter group. The Neb-SURF600 group showed slightly lower mean PaO_2_ values than Neb-SURF200 and Neb-SURF400 groups at 180 min.

Surfactant depletion by BALs was associated with severe hypercapnia and acidosis in all groups. Hypercapnia exacerbated even further in the nCPAP group, whereas surfactant-treated groups showed a downward trend of mean PaCO_2_ values (Fig. 2b). However, a significant reduction of PaCO_2_ in comparison to the nCPAP group was only observed for Inst-SURF, Neb-SURF200 and Neb-SURF400 groups (*P* < 0.05). Severe acidosis (pH < 7.2) persisted in the nCPAP group for the whole 180 min follow up period, with pH values that were significantly lower compared to any surfactant-treated group (Fig. 2c).

**Fig. 3:**
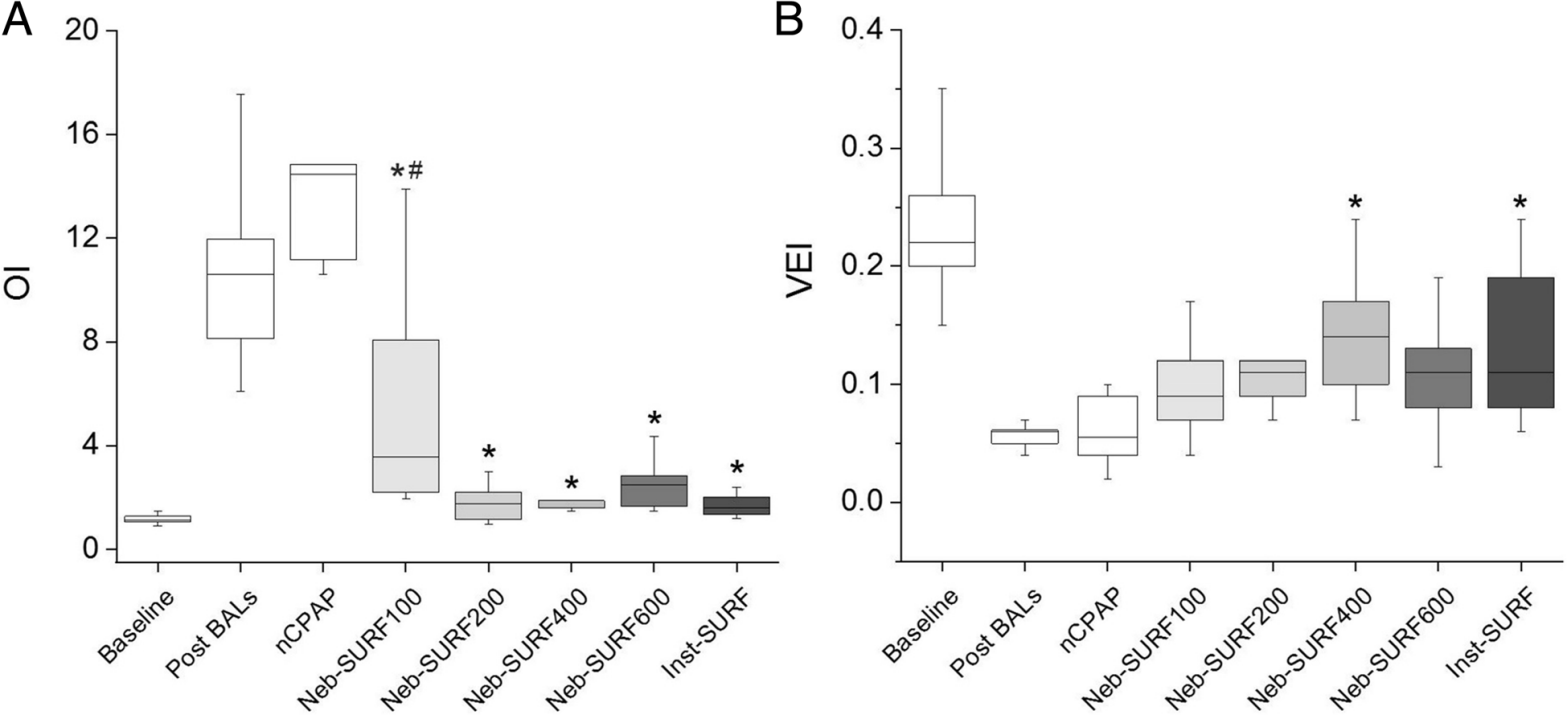
Box-plots showing (**a**) the oxygenation index (OI) and (**b**) ventilation efficacy index (VEI) at baseline (from all animals), after inducing a respiratory distress (Post BALs, from all animals) and 180 min after treatment with just nasal continuous positive pressure ventilation (nCPAP), with different doses of nebulized surfactant (Neb-SURF100, Neb-SURF200, Neb-SURF400, Neb-SURF600) or with intratracheal surfactant (Inst-SURF). The boxes encompass the 25–75 percentiles. The horizontal line within the boxes represents the median. The whiskers indicate the maximum and minimum values observed for each group. * *P* vs. nCPA *P* < 0.01; ^#^ *P* vs. Inst-SURF < 0.05

At baseline, OI was below 1.5 and the VEI above 0.15 in all study subjects. After BALs, mean OI increased to 10.84 ± 2.94 and the VEI dropped to 0.06 ± 0.01 (Fig. 3). At the end of the experimental period, OI was significantly lower in all surfactant-treated groups compared with the nCPAP group (*P* < 0.01). At 180 min, VEI remained low in the nCPAP group but increased in all surfactant-treated groups. However, a significant improvement of VEI compared to the nCPAP group was only observed for Inst-SURF and Neb-SURF400 groups (*P* < 0.05).

### Pulmonary mechanics

**Fig. 4:**
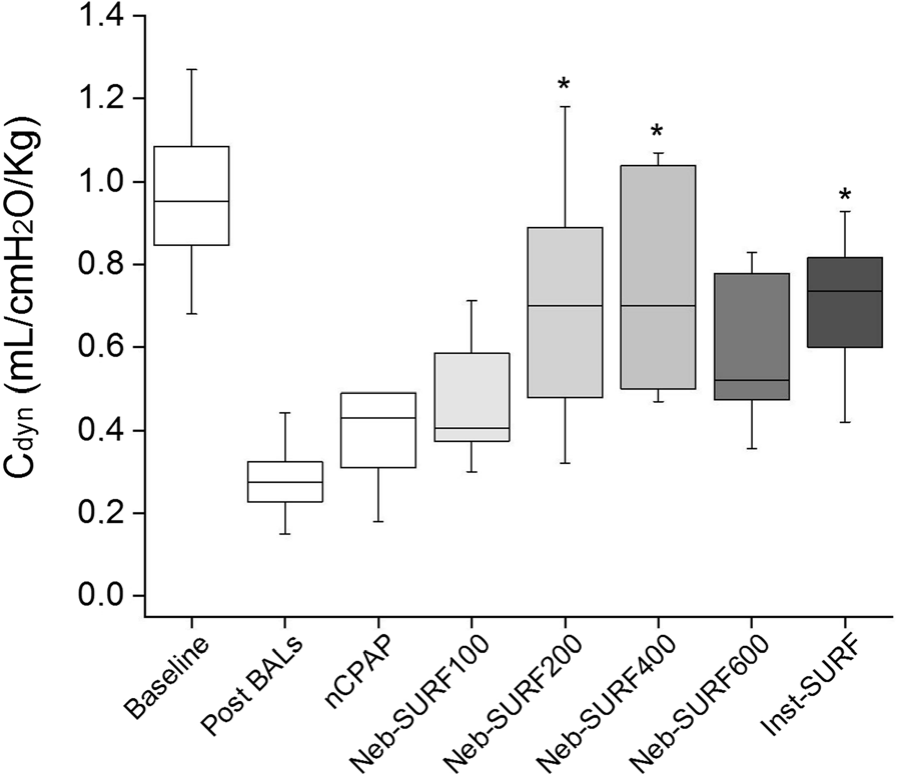
Box-plots showing dynamic compliance (C_dyn_) at baseline (from all animals), after inducing a respiratory distress (Post BALs, from all animals) and 180 min after treatment with just nasal continuous positive pressure ventilation (nCPAP), with different doses of nebulized surfactant (Neb-SURF100, Neb-SURF200, Neb-SURF400, and Neb-SURF600) or with intratracheal surfactant (Inst-SURF). The boxes encompass the 25–75 percentiles. The horizontal line within the boxes represents the median. The whiskers indicate the maximum and minimum values observed for each group. The whiskers indicate the maximum and minimum values observed for each group. * *P* vs. nCPAP < 0.05

C_dyn_ significantly dropped in all groups after the induction of respiratory failure (Fig. 4). Mean C_dyn_ values measured after 180 min were relatively low in nCPAP (0.38 ± 0.06 mL/cmH_2_O/kg) and Neb-SURF100 (0.46 ± 0.05 mL/cmH_2_O/kg) groups. Conversely, mean C_dyn_ values significantly increased in the Inst-SURF, Neb-SURF200 and Neb-SURF400 groups after 180 min of non-invasive ventilation compared to the nCPAP group (*P* < 0.05).

### Alveolar DSPC quantification

The mean amount of exogenous DSPC in the Neb-SURF100 was 33-fold lower compared to the Inst-SURF group. Conversely, the Neb-SURF200 group registered an amount of exogenous DSPC 3.7-fold lower compared to the Inst-SURF group. Neb-SURF400 and Neb-SURF600 groups showed the highest mean intrapulmonary DSPC amounts just 1.8- and 1.2-fold lower compared to the animals which received intratracheal surfactant. These groups, however, showed a high inter-animal variability (Additional file 1: Figure S2).

## Discussion

We investigated the feasibility of delivering undiluted nebulized surfactant with the customized eFlow Neos vibrating-membrane nebulizer system under realistic neonatal ventilation conditions. Laser diffraction studies demonstrated suitable aerosol characteristics for inhalation in terms of particle size distribution and fine particle fraction. The theoretical surfactant LD determined in vitro in a rational neonatal CPAP circuit, which included the simulated breathing pattern of a preterm infant with mild RDS, reached notable values for neonatal standards, as high as 19.8% of the nominal dose. Further, a dose-response study conducted in spontaneously-breathing lung-lavaged rabbits revealed a significant improvement of arterial oxygenation following treatment with nebulized surfactant. Particularly, the groups of animals treated with 200 mg/kg and 400 mg/kg of nebulized *poractant alfa* achieved an equivalent pulmonary response as the group of animals treated with instilled surfactant (200 mg/kg).

The concept of nebulized surfactant dates back to 1964 and was indeed one of the first attempts to treat preterm infants with RDS [54, 55]. Nevertheless, aerosol technology and lung deposition mechanisms were poorly understood at that time, which moved the field to the development of intratracheal instillation protocols [56, 57]. Since then, surfactant replacement therapy has undergone a constant evolution towards a less invasive administration [4, 9, 58]. At present, the LISA method is experiencing worldwide clinical appraisal. This technique significantly reduces the need for mechanical ventilation and oxygen supplementation, and may as well reduce the incidence of BPD [59]. However, it still involves the acute fluid load of the infant’s airway, which may lead to transient airway obstruction, surfactant reflux, and hemodynamic imbalance [16].

In this study, we intended to develop a neonate-focused surfactant nebulization strategy, taking into consideration not only the particular characteristics of preterm infants but also the relatively high viscosity of surfactant. Finner et al. reported marked variability in surfactant dose output among single Aeroneb Pro (Aerogen) vibrating-membrane nebulizer units during a pilot clinical study evaluating the potential of aerosolized surfactant for the prevention of RDS [34]. The authors attributed the variability in dose output to higher viscosity of surfactant compared to medications which are routinely delivered as aerosols. They discouraged the use of this device in combination with surfactant. Additional studies with nebulized surfactants have reported clogging of the pores of the vibrating membrane of the nebulizer, requiring surfactant dilution before nebulization [30, 60]. In the present study, the customized eFlow-Neos nebulizer system was able to produce respirable aerosols of *poractant alfa*, a highly concentrated surfactant, which is formulated at around 80 mg of phospholipids/mL. It is noteworthy that particle size distribution, as well as the output rate of the single customized eFlow-Neos nebulizing units, showed consistent results. We used new nebulizer units for each in vivo experiment, as well as 5 independent units for the in vitro study, overall analyzing the performance of 41 nebulizer units. All units showed a consistent output rate and performed well with undiluted *poractant alfa* (Additional file 1: Figure S3).

We further tested the customized eFlow-Neos nebulizer in a rational neonatal CPAP circuit. In order to maximize the surfactant LD, we placed the nebulizer between the Y piece and the nasal prongs. Surfactant aerosols were thus generated close to the nares of the PrINT model. This cast resembles the upper airways (nose-throat) of a premature infant born at a gestational age of 32 weeks and is a useful tool to estimate the LD of nebulized medications intended for delivery to preterm infants [41]. Minocchieri et al. investigated the theoretical LD of inhaled budesonide generated with another vibrating-membrane nebulizer prototype (e-Flow, PARI GmbH Starnberg, Germany). They found that the LD dropped from around 62 to 9% of the nominal dose if the bias flow-rate was increased from 1 L/min to 10 L/min [41]. In comparison to their set up, we used humidified air, a bias flow of 5 L/min, a CPAP support of 5 cmH_2_O, and simulated the breathing pattern of a neonate with mild RDS. Under such a challenging configuration, the minimal and maximal surfactant LDs were 10.2 and 19.8% of the nominal dose (200 mg/kg), respectively, corresponding to surfactant doses of 20.4 mg/kg and 39.6 mg/kg. Since individual nebulizer units showed a consistent performance in terms of surfactant output and particle size, we assume that the variability in LD observed for each single experiment may be rather related to slight changes in the positioning of the components of the circuit (e.g. small changes in the angle of the prongs) or to slight variations of the air-flow of the circuit from experiment to experiment. Nevertheless, considering that intrapulmonary doses as low as 2 mg/kg of nebulized surfactant have been associated with a significant improvement of lung mechanics [22], we performed a dose-response study using four escalating nominal doses of *poractant alfa* (dose-range 100–600 mg/kg).

The in vivo study was conducted in lung-lavaged, spontaneously-breathing rabbits managed with nCPAP [48]. A particular advantage of this model is that the animals do not spontaneously recover from the respiratory distress if they are just supported with non-invasive ventilation [48, 49]. Therefore, any improvement of oxygenation or lung mechanics observed over time can be attributed to lung deposition of active exogenous surfactant. A surfactant dose of 100 mg/kg produced already a significant improvement of arterial oxygenation. According to our in vitro study, a nominal dose of 100 mg/kg would achieve a LD of 13.7 mg/kg. Nevertheless, this dose did not suffice to revert the respiratory distress. On the other hand, nebulization of 200 and 400 mg/kg of surfactant was associated with a significant improvement of arterial oxygenation, respiratory indices, and pulmonary mechanics, achieving a pulmonary response at the same level of that observed for animals treated with an intratracheal bolus of 200 mg/kg of surfactant. It is noteworthy that such an improvement was achieved administering nebulized surfactant to spontaneously-breathing animals managed with nCPAP. These results are highly encouraging from a clinical point of view.

As a proxy of surfactant lung deposition, we determined the amount of exogenous DSPC in three animals of each surfactant-treated group. Although the limited sample size precluded a sound statistical test, a clear dose-dependent trend could be identified across the groups of animals treated with nebulized surfactant. Linner et al. determined by means of gamma scintigraphy that approximately 90% of the instilled surfactant reaches the lungs following intratracheal admnistration [60]. Therefore, we normalized the exogenous DSPC amount of the groups of animals treated with nebulized surfactant to the DSPC amount determined for the Inst-SURF group. The relative exogenous DSPC quantified in the BALs of Neb-SURF200 was almost 4.0-fold lower compared to the BALs of the Inst-SURF group, even though the elicited pulmonary response was equivalent in both groups. We speculate that a more uniform intrapulmonary spreading of surfactant after nebulization might have accounted for the similar pulmonary outcome observed for both groups. Our data also suggest that a nominal surfactant dose of 400 mg/kg may be of further advantage because it provides a good short term pulmonary response but also contributes to increase the intrapulmonary surfactant pool. Surprisingly, a further increase of the nominal dose to 600 mg/kg did not produce additional benefits in terms of gas exchange or pulmonary mechanics. This was an unexpected result. In other animal models, high surfactant doses have been associated with increased inflammation and neutrophil migration [61] as well as with a reduction of the antibacterial defenses [62]. Nevertheless, we suspect that in this study the continuous nebulization of 600 mg/kg of surfactant may have produced surfactant accumulation in the airways, which would partly explain lower PaO_2_ and slightly higher PaCO_2_ values of this groups compared to Neb-SURF200 and Neb-SURF400 groups. Unfortunately, we could not determine the pulmonary distribution of nebulized surfactant in this work and we acknowledge this as a limitation of the study.

## Conclusion

We have carried out a complete preclinical assessment of the customized eFlow-Neos nebulizer operated with *poractant alfa* for the treatment and prevention of RDS. The nebulizer efficiently generated respirable aerosols out of undiluted surfactant, yielding an estimated mean lung deposition of 13.7% in vitro. Benchmark experiments settled the basis to implement a dose-response study in spontaneously-breathing rabbits with severe respiratory distress. Nominal surfactant doses of 200 mg/kg and 400 mg/kg delivered with the customized eFlow neos nebulizer during nCPAP significantly improved oxygenation, respiratory indices, and lung compliance, eliciting a pulmonary response equal to the one observed for the group of animals treated with an intratracheal bolus of 200 mg/kg of surfactant. The lung improvement induced by surfactant nebulization was achieved with a lower intrapulmonary surfactant dose compared to the InSurE approach. The neonate-focused surfactant nebulization strategy described in this work is currently being evaluated in Phase II clinical trial (EUDRACT No.:2016–004547-36).

## Additional file

Additional file 1: **Table S1.** Baseline and post lung injury (Post-BAL) characteristics of surfactant-depleted adult rabbits treated with nasal continuous positive pressure ventilation (nCPAP), with intratracheal surfactant (Inst-SURF), or with different doses of nebulized surfactant (Neb-SURF100, Neb-SURF200, Neb-SURF400, and Neb-SURF600. **Figure S1.** PrINT cast with silicon-coated nostrils. **Figure S2.** Nebulizer output assessment. (DOCX 239 kb)

## Data Availability

The datasets used and/or analysed during the current study are available from the corresponding author on reasonable request. They are not immediately pubblicaly available because they have been obtained by a private-funded research activity.

## Abbreviations

BAL: Broncho-Alveolar Lavage
BPD: Broncho-Pulmonary Dysplasia
CPAP: Continuous Positive Airway Pressure
DSPC: Disaturated-Phosphatidylcholine
FiO_2_: Fraction of Inspired Oxygen
FPF: Fine Particle Fraction
GSD: Geometric Standard Deviation
HPLC: High Pressure Liquid Chromatography
LD: Lung Dose
LISA: Less Invasive Surfactant Administration
MAP: Mean Airway Pressure
MMD: Mass Median Diameter
nCPAP: nasal Continuous Positive Airway Pressure
OI: Oxygenation Index
PaCO2: Arterial carbon dioxide partial pressure
PaO2: Arterial Oxygen Partial Pressure
PEEP: Positive End-expiratory Pressure
PIP: Peak Inspiratory Pressure
RDS: Respiratory Distress Syndrome
RH: Relative Humidity
RR: Respiratory Rate
VEI: Ventilation Efficacy Index
V_T_: Tidal Volume
