# Supplementary material for: From bench to bedside: in vitro and in vivo evaluation of a neonate-focused nebulized surfactant delivery strategy
Source: Respir Res. 2019 Jul 2;20:134. doi: 10.1186/s12931-019-1096-9 (PMC6604359; doi:10.1186/s12931-019-1096-9)
Supplement: Supplementary file 1 — Table S1. Baseline and post lung injury (Post-BAL) characteristics of surfactant-depleted adult rabbits treated with nasal continuous positive pressure ventilation (nCPAP), with intratracheal surfactant (Inst-SURF), or with different doses of nebulized surfactant (Neb-SURF100, Neb-SURF200, Neb-SURF400, and Neb-SURF600. Figure S1. PrINT cast with silicon-coated nostrils. Figure S2. Nebulizer output assessment. (DOCX 239 kb) [file 12931_2019_1096_MOESM1_ESM.docx]

**FROM BENCH TO BEDSIDE: *IN VITRO* AND *IN VIVO* EVALUATION OF A NEONATE-FOCUSED NEBULIZED SURFACTANT DELIVERY STRATEGY**

Bianco F^1^, Ricci F^1^, Catozzi C^1^, Murgia X^2^, Schlun M^3^, Bucholski A^3^, Hetzer U^3^, Bonelli S^1^, Lombardini M^1^, Pasini E^1^, Nutini M^1^, Pertile M^1^, Minocchieri S^4^, Simonato M^5^, Rosa B^1^, Pieraccini G^6^, Moneti G^6^, Lorenzini L^7^, Catinella S^1^, Villetti G^1^, Civelli M^1^, Pioselli B^1^, Cogo P^8^, Carnielli V^9^ Dani C^10^*, Salomone F^1^

*^1^Department of Preclinical Pharmacology, R&D, Chiesi Farmaceutici S.p.A., Parma, Italy.*

*^2^Scientific Consultancy, Saarbrücken, Germany.*

*^3^PARI Pharma GmbH, Starnberg, Germany.*

*^4^Division of Neonatology, Cantonal Hospital Winterthur, Winterthur, Switzerland.*

*^5^Pediatric Research Institute “Città della Speranza” Padova, Italy*

*^6^Mass Spectrometry Center (CISM), Polo Biomedico, Careggi University Hospital of Florence, Florence, Italy.*

*^7^Health Science and Technologies Interdepartmental Center for Industrial Research (HST-ICIR), University of Bologna, Bologna, Italy.*

*^8^Division of Pediatrics, Department of Medicine, University of Udine*

*^9^Polytechnic University of Marche and Azienda Ospedaliero-Universitaria Ospedali Riuniti, Ancona, Italy.*

*^10^Department of Neurosciences, Psychology, Drug Research and Child Health, Careggi University Hospital of Florence, Florence, Italy.*

*Corresponding author:

Carlo Dani, MD, Division of Neonatology,

Careggi University Hospital, University of Florence School of Medicine,

Viale Morgagni, 85 Firenze, Italy (e-mail: cdani@unifi.It).

**SUPPLEMENTARY INFORMATION**

**SUPPLEMENTARY TABLE 1**. Baseline and post lung injury (Post-BAL) characteristics of surfactant-depleted adult rabbits treated with nasal continuous positive pressure ventilation (nCPAP), with intratracheal surfactant (Inst-SURF), or with different doses of nebulized surfactant (Neb-SURF100, Neb-SURF200, Neb-SURF400, and Neb-SURF600.

| **Group** | **Birth Weight**  **(kg)** | **Number of BALs** | **Baseline PaO_2_ (mmHg)** | **Post-BALs PaO_2_ (mmHg)** | **Baseline**  **Cdyn (mL/cmH_2_O/kg)** | **Post-BALs**  **Cdyn (mL/cmH_2_O/kg)** |
| --- | --- | --- | --- | --- | --- | --- |
| **nCPAP** | 1.72 ± 0.1 | 7.33 ± 1 | 473 ± 19 | 95 ± 11 | 0.88 ± 0.05 | 0.20 ± 0.02 |
| **Inst-SURF** | 1.70 ± 0.1 | 5.10 ± 1 | 468 ± 13 | 70 ±17 | 1.09 ± 0.02 | 0.31 ± 0.04 |
| **Neb-SURF100** | 1.80 ± 0.1 | 7.44 ± 1 | 456 ± 14 | 91 ± 7 | 0.93 ± 0.06 | 0.28 ± 0.04 |
| **Neb-SURF200** | 1.71 ± 0.1 | 5.78 ± 1 | 472 ± 11 | 93 ± 8 | 1.07 ± 0.09 | 0.34 ± 0.02 |
| **Neb-SURF400** | 1.76 ± 0.1 | 6.22 ± 1 | 478 ± 8 | 101 ±10 | 1.02 ± 0.04 | 0.22 ± 0.02 |

BAL, Broncho-Alveolar Lavage; PaO_2_, Arterial Oxygen Partial Pressure; C_dyn_, Dynamic compliance.

**SUPPLEMENTARY FIGURE 1**. PrINT cast with silicon-coated nostrils**.**

*
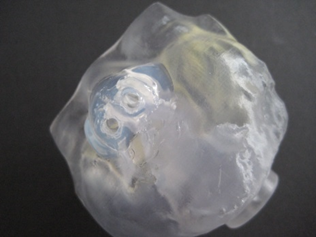
*

The infant nose-throat cast was 3D printed (1zu1 prototypen, Dornibirn, Austria) as a solid substance (material: DSM water clear ultra 10122). The nose area was silicon-coated to achieve a tight connection between cast and prongs.

**SUPPLEMENTARY FIGURE 2.** Nebulizer output assessment.


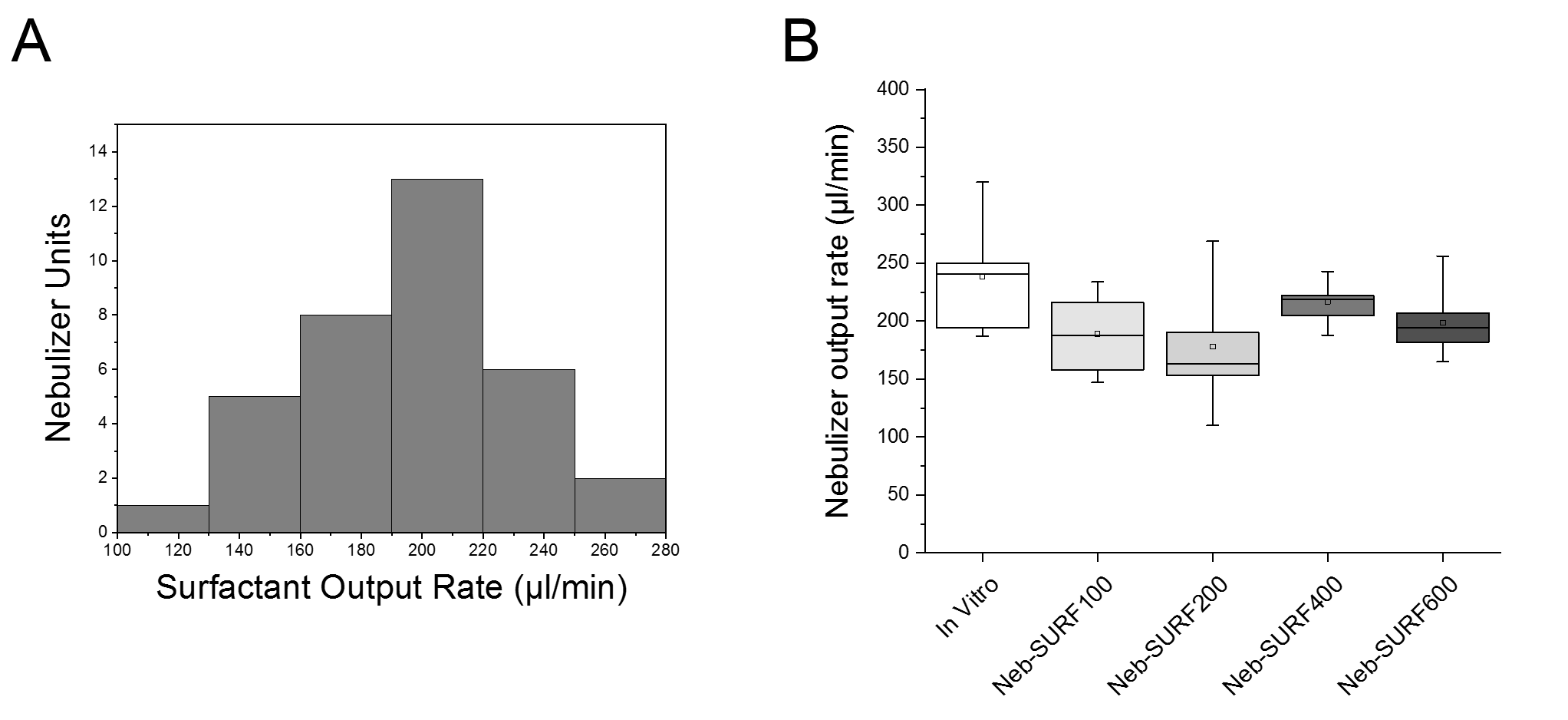


In total 41 independent nebulizer units were used in the present study. (A) Shows the distribution of the output rate of all devices. In (B) the box-plot shows the output rate as a function of the treatment group. The 5 nebulizers used in the *in vitro* study are also represented in the graph. The boxes encompass the 25-75 percentile. The horizontal line within the box represents the median, whereas the small square represents the mean. The whiskers indicate the maximum and minimum values for each group.
